# Simplified Synthesis of the Amine-Functionalized Magnesium Ferrite Magnetic Nanoparticles and Their Application in DNA Purification Method

**DOI:** 10.3390/ijms241814190

**Published:** 2023-09-16

**Authors:** Ágnes M. Ilosvai, Tímea B. Gerzsenyi, Emőke Sikora, Lajos Harasztosi, Ferenc Kristály, Béla Viskolcz, Csaba Váradi, Emma Szőri-Dorogházi, László Vanyorek

**Affiliations:** 1 Institute of Chemistry, Faculty of Materials and Chemical Engineering, University of Miskolc, 3515 Miskolc, Hungary; maria.agnes.ilosvai@uni-miskolc.hu (Á.M.I.); emoke.sikora@uni-miskolc.hu (E.S.); bela.viskolcz@uni-miskolc.hu (B.V.); 2 Higher Education and Industrial Cooperation Centre, University of Miskolc, 3515 Miskolc, Hungary; timea.gerzsenyi@uni-miskolc.hu (T.B.G.); csaba.varadi@uni-miskolc.hu (C.V.); 3 Department of Solid-State Physics, Faculty of Science and Technology, University of Debrecen, 4010 Debrecen, Hungary; lajos.harasztosi@science.unideb.hu; 4 Institute of Mineralogy and Geology, Faculty of Earth and Environmental Sciences and Engineering, University of Miskolc, 3515 Miskolc, Hungary; ferenc.kristaly@uni-miskolc.hu

**Keywords:** magnetic nanoparticles, amine-functionalized magnesium ferrite, fast microwave synthesis, DNA binding

## Abstract

For pathogens identification, the PCR test is a widely used method, which requires the isolation of nucleic acids from different samples. This extraction can be based on the principle of magnetic separation. In our work, amine-functionalized magnesium ferrite nanoparticles were synthesized for this application by the coprecipitation of ethanolamine in ethylene glycol from Mg(II) and Fe(II) precursors. The conventional synthesis method involves a reaction time of 12 h (MgFe_2_O_4_-H&R MNP); however, in our modified method, the reaction time could be significantly reduced to only 4 min by microwave-assisted synthesis (MgFe_2_O_4_-MW MNP). A comparison was made between the amine-functionalized MgFe_2_O_4_ samples prepared by two methods in terms of the DNA-binding capacity. The experimental results showed that the two types of amine-functionalized magnesium ferrite magnetic nanoparticles (MNPs) were equally effective in terms of their DNA extraction yield. Moreover, by using a few minutes-long microwave synthesis, we obtained the same quality magnesium ferrite particles as those made through the long and energy-intensive 12-h production method. This advancement has the potential to improve and expedite pathogen identification processes, helping to better prevent the spread of epidemics.

## 1. Introduction

Nanomaterials are continuously increasing in popularity for their advantageous properties in catalysis [1], carbon dioxide sequestration [2,3,4] and biological applications [5]. Among the various nanomaterials, magnetic nanoparticles (MNPs) are used for biological applications, such as magnetic resonance imaging [6], hyperthermia treatment [7], drug delivery [8] or the isolation of macromolecules [9], like deoxyribonucleic acid (DNA). Some of the advantageous properties of the magnetic nanomaterials in these applications are biocompatibility, low or zero toxicity, cost-effectiveness, superparamagnetic properties, a high binding capacity and straightforward synthesis [10]. Generally, DNA isolation can be achieved by liquid–liquid phase extraction methods, like alkaline lysis and phenol-chloroform extraction, or with solid–liquid phase extraction, such as silica cartridge-based or magnetic nanoparticle-based isolation [11]. In the case of purification and separation techniques, MNPs are more frequently utilized due to the cost-effective nature of the method, its practicality, and lower toxicity compared to conventional isolation methods [12,13].

From the vast available literature, some are especially important for our research on DNA isolation using magnetic nanoparticles. For example, Prodělalová et al. [14] used cobalt ferrite MNPs for genomic DNA isolation from different dairy products. Modified MNPs were also used for nucleic acid isolation applications [15], where the Al^3+^- and iminodiacetic acid-modified surfaces of ferrite nanoparticles have an increased affinity to DNA. Other approaches like silica- or amine-functionalized MNPs have also been developed and demonstrated that these MNPs are also good solid-phase candidates in the extraction of plasmid or genomic DNA [9,16,17,18,19]. In the case of iron oxide MNPs, it has been observed that a higher density of amino groups on the particle surface leads to improved DNA-binding efficiency [20,21]. On the other hand, numerous papers describe that amino-modified magnetic materials can adsorb DNA molecules at low salinity or neutral pH conditions [22,23,24,25,26].

Plasmid DNA (pDNA) is an extrachromosomal, circular, double-stranded DNA molecule, usually found in a small nucleic acid compartment in bacterial, archaeal or some eukaryotic organisms. The chemical composition of the plasmids does not differ from the genomic DNA [27]; therefore, plasmids are often used as a tool for molecular cloning and gene delivery systems. For such downstream applications, the quantity and quality of the purified pDNA must be determined and meet some criteria. The absorbance ratio measured at 260 nm and 280 nm is used to assess the purity (quality) of the isolated DNA, and according to the literature, a ratio of 1.7–2.0 is accepted as a pure DNA solution. A considerably lower ratio (below 1.6) may indicate contamination with protein, phenol or other reagents that absorb at 280 nm and can negatively affect further processes. On the other hand, the presence of RNA can lead to an increase in the ratio value [28]. Generally, the DNA isolation methods that provide high quality and an adequate quantity of the extracted plasmid or genomic DNA are at the center of specific scientific interest.

Nanoparticles that are used in molecular biological or bioanalytical separation processes have superparamagnetic properties (e.g., magnetite, maghemite and alkaline earth or transition metal ferrites). These ferrites are spinel-type metal oxides with the chemical composition MFe_2_O_4_, where M denotes divalent metal ions [29], such as Mn^2+^, Ni^2+^, Co^2+^, Mg^2+^, Zn^2+^ and Cd^2+^ [7,30]. These ferrite nanoparticles may differ not only in their composition but also in their preparation techniques and the corresponding synthesis time.

In the literature, there are examples of longer and energetically less efficient methods, as well as protocols that propose shorter synthesis times to produce magnetic nanoparticles. Chattharika et al. reported a 12-h coprecipitation synthesis method (abbreviated as H&R due to its heating and refluxing steps) for amine-functionalized magnesium ferrite magnetic nanoparticle (MgFe_2_O_4_ MNP) preparation and characterized its adsorption properties using Congo red [31]. Vishwas et al. published a 4-h coprecipitation protocol for cobalt ferrite nanoparticles used in photocatalysis [32]. Yadav and co-workers have described a simple 60-min-long sonochemical method for the synthesis of gadolinium-doped cobalt ferrite nanoparticles [33]. Compared to these procedures, microwave-assisted synthesis (MW) can be an even faster way to produce large amounts of magnetic nanoparticles. The advantages of MW synthesis include fast volume heating, high reaction rates, size and shape control by adjusting the reaction parameters and energy efficiency. Moreover, MW synthesis enables homogenous heating, minimizing the thermal gradients and providing uniform nucleation and growth conditions, resulting in magnetic nanoparticles with a consistent size distribution [34]. For example, Ayyıldız and co-workers applied MW synthesis for 35 min to prepare cobalt ferrite nanoparticles, which were then used to extract Pb ions in rooibos (*Aspalathus linearis*) tea [35], while Abhishek et al. produced titanium ferrite MNPs with a 15-min MW synthesis. They found that varying the synthesis time could improve the magnetic properties of the particles [36].

Based on the literature examples, the aim of our work was to prepare amine-functionalized magnesium ferrite nanoparticles by using both the classical 12-h coprecipitation method (MgFe_2_O_4_-H&R MNP) and 4-min microwave treatment (MgFe_2_O_4_-MW MNP). The physicochemical properties of the resulting particles (such as particle morphology, specific surface area, size, phase composition, surface polarity and dispersibility) were characterized by high-resolution transmission electron microscopy (HRTEM), selected area diffraction (SAED), X-ray powder diffraction (XRD), Fourier-transform infrared spectroscopy (FTIR) and laser-Doppler electrophoresis (zeta potential) measurements. The DNA-binding ability was also investigated; to the best of our current scientific knowledge, magnesium ferrite magnetic nanoparticles have not been used for DNA isolation before. The DNA-binding ability and DNA-binding efficiency of the synthesized MNP samples were compared, and the isolation efficiency and isolated pDNA were tested using molecular biological downstream processes. The DNA extraction efficiency was also compared to that of a commercially available MNP-based isolation kit in terms of the DNA yield capacity.

## 2. Results and Discussion

### 2.1. Surface Area Measurements

A basic feature of the MNPs in DNA isolation applications is that the DNA reversibly adsorbs to the surface of amine-functionalized MNPs, where the specific surface area (SSA) has a crucial role. Thus, CO_2_ adsorption–desorption measurements were carried out at 273 K based on the Dubinin–Astakhov (DA) method to determine the SSA. The surface areas were 86.3 m^2^ g^−1^ (MgFe_2_O_4_-MW) and 54.4 m^2^ g^−1^ (MgFe_2_O_4_-H&R)—the difference reflecting the importance of the synthesis route. MgFe_2_O_4_ samples with similar specific surface areas (SA_BET_: 47 m^2^ g^−1^ and 56.5 m^2^ g^−1^) were reported previously using a coprecipitation method by applying long-time reflux [31,37].

### 2.2. Particle Size Distribution

**Figure 1:**
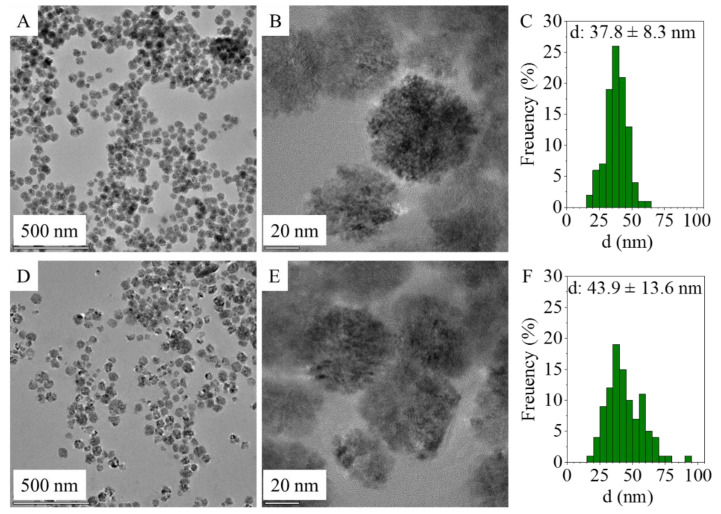
HRTEM images of the MgFe_2_O_4_-H&R (**A**,**B**) and MgFe_2_O_4_-MW (**D**,**E**) and particle size distribution of MgFe_2_O_4_-H&R (**C**) and MgFe_2_O_4_-MW (**F**).

An increased SSA can be obtained by using ultrafine particle sizes, highlighting the importance of nanoparticles since the quantity of anchored DNA depends on the accessible surface area of the adsorbent [38]. In this sense, the determination of the particle size distribution is important, and it was acquired from HRTEM images of the amine-functionalized MgFe_2_O_4_ samples using ImageJ image analyser software (Figure 1A,B,D,E). Based on the particle diameters, size distribution histograms were built and compared with other magnesium ferrites prepared by different methods. In the case of all the samples acquired from the HRTEM images, similar spherical ferrite nanoparticles were visible despite the different synthesis methods (Figure 1 A,B,D,E). These aggregated spheres build up from individual 4–8 nm sized nanoparticles (SI Figure 1A,B).

The average particle size of the MgFe_2_O_4_-MW spheres (aggregates) is 43.9 ± 13.6 nm and is in a wide distribution range (Table 1) based on the standard deviation and while considering the interquartile range. A similar distribution was observed in the case of the amine-functionalized MgFe_2_O_4_-H&R nanoparticles, where the average particle size was 37.8 ± 8.3 nm (Figure 1C,F). During the synthesis of the amine-functionalized MgFe_2_O_4_-MW nanospheres, higher diameters developed: 43.9 ± 13.6 nm (Table 1 and Figure 1F). A total of 10% of the MgFe_2_O_4_-MW MNPs had sizes exceeding 61.8 nm, while 10% of the MgFe_2_O_4_-H&R MNPs were larger than 47.3 nm. In the case of the two ferrite samples, the mean particle sizes are quite close to the corresponding median values, which is a characteristic of the normal distribution.

### 2.3. XRD Characterization of Amine-Functionalized Magnesium Ferrite Nanoparticles

**Figure 2:**
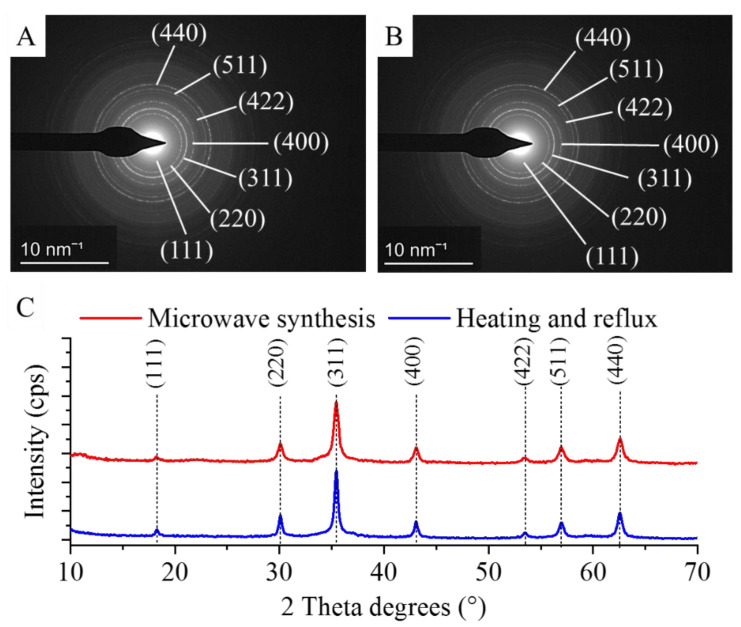
SAED image of the MgFe_2_O_4_-H&R (**A**) and MgFe_2_O_4_-MW (**B**) particles and X-ray powder diffraction of the MgFe_2_O_4_-H&R and MgFe_2_O_4_-MW nanopowders (**C**).

The individual nanospheres in both types of amine-functionalized MgFe_2_O_4_ MNP samples were examined by selected area diffraction (SAED) during the HRTEM analysis, and the measured d-spacings were matched with the d-values in the diffraction databases (Figure 2A,B). After identifying the individual particles, it was necessary to examine both types of nanopowder samples using the X-ray powder diffraction (XRD) method. XRD is the only reliable technique for the bulk-phase identification, quantification and size-shape characterization required for our samples, especially since non-ferrite phases might occur in the samples as technological residues. The XRD pattern of the samples (Figure 2C) revealed peaks of only magnesium ferrite, located at (2Th/hkl) 18.2°/(111), 30.1°/(220), 35.4°/(311), 43.1°/(400), 53.4°/(422), 56.9°/(511) and 62.6°/(440), matching the PDF 36–0398 card. Other phases as residue were not detected; therefore, it can be stated that the two proposed synthesis methods are equally suitable for obtaining pure spinel-phase MNPs.

### 2.4. FTIR Measurements

**Figure 3:**
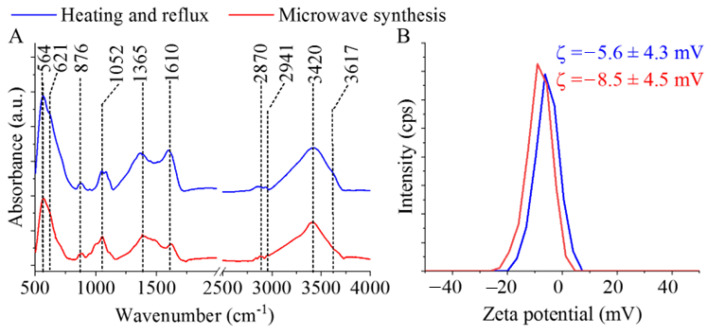
FTIR spectra of the MgFe_2_O_4_-H&R and MgFe_2_O_4_-MW (**A**), as well as zeta potential distribution diagrams and average zeta potential values of these ferrites (**B**).

The identification of surface functional groups—best detected by FTIR measurements—is very important regarding the dispersibility of MNPs in polar solvents. Two characteristic bands are observed at the 564 cm^−1^ and 621 cm^−1^ wavenumbers, which are assigned to the intrinsic stretching vibration modes of the metal–oxygen bonds at the octahedral and tetrahedral sites [39] (Figure 3A). Other bands found on the recorded spectra are at 876 cm^−1^ and 1052 cm^−1^, assigned to the νC–O and the νC-N vibrations, which originate from the hydroxyl and carboxyl groups, where the νC-N vibrations developed with the presence of amine functional groups. Other absorption bands are found at 1365 cm^−1^, ascribed to the C–O vibrations and the C–N stretching vibrations, respectively. The stretching vibration band of the N–H bonds overlaps with the vibration band of the -OH groups. Another band was identified as a carbon-related bond at the 1610 cm^−1^ wavenumber, belonging to the βN–H (bending vibration mode) of the free amine functional groups. The symmetric and asymmetric stretching vibration of the aliphatic and aromatic C–H bonds resulted in absorption at 2870 cm^−1^ and 2941 cm^−1^, which can be explained by the adsorbed organic molecules (EG and EA) on the surface [40,41]. The stretching vibration bands of the hydroxyl and amine groups overlap and result in a broad region at 3420 cm^−1^ and 3617 cm^−1^. Zeta potential (ZP) measurements were carried out at an ambient pH in distilled water (pH: 6). The surface hydroxyl groups are partially found in a deprotonated form, resulting in negative ZPs (-5.6 ± 4.3 mV and -8.5 ± 4.5 mV). Next to the -OH groups, -NH_2_ functional groups are found on the nanoparticles, which can become protonated when the pH decreases, leading to the appearance of positive charges (-NH^3+^). Due to the positive charges formed as a result of the protonation of the amine groups, negatively charged DNA can form an electrostatic interaction with the magnetic nanoparticles. By increasing the pH (adding an elution buffer), the positive charges on the surface can be reduced. Then, the deprotonation of the -OH groups result in a negative ZP, thus eliminating the electrostatic interaction between the DNA and nanoparticles. We must also take into account that other interactions can develop between DNA and ferrite nanoparticles. In addition to the electrostatic interactions, other adsorption mechanisms can also take place, as the nitrogen-containing bases in DNA can strongly coordinate with the surface of the metal oxide, i.e., the Fe_3_O_4_ surface [42]. Owing to this, the magnetic nanoparticles can be coprecipitated by DNA molecules.

Due to the surface hydroxyl and amine functional groups, hydrogen bonds can be formed between the nanoparticles and DNA molecules [43,44]. Such interaction sites enable the DNA to bind reversibly to the magnetic nanoparticles during the extraction process, and thus, by changing the buffer medium, it can be easily separated from the unwanted cell-forming macromolecules [43,44].

### 2.5. Magnetization Measurements

**Figure 4:**
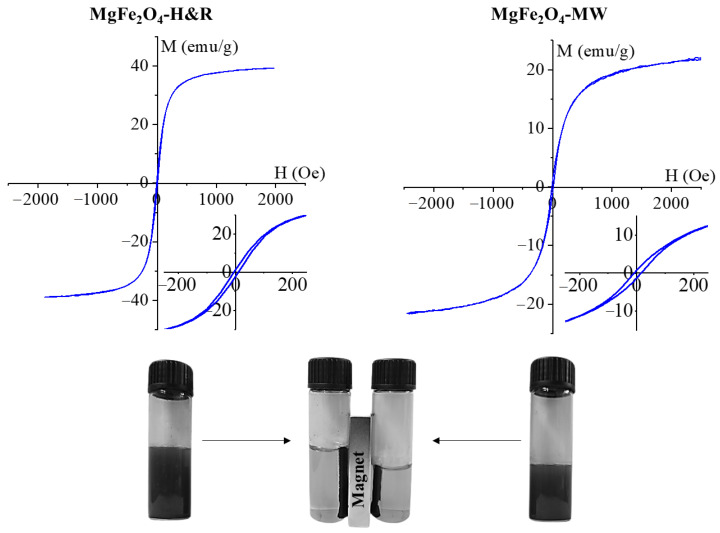
Magnetization curves of the MgFe_2_O_4_-H&R and MgFe_2_O_4_-MW samples, with the narrow hysteresis loops in higher scale resolution (insets). The photo at the bottom demonstrates the dispersibility in a polar solvent and the magnetic separability of the ferrite nanoparticles.

The magnetization curve of the amine-functionalized MgFe_2_O_4_ samples was measured at room temperature using a vibrating-sample magnetometer (VSM). The magnetic saturation (Ms) reached 36 emu/g in the case of MgFe_2_O_4_-H&R, as shown in Figure 4A. A lower Ms value (21 emu/g) was measured in the MgFe_2_O_4_-MW sample (Figure 4B). For the MgFe_2_O_4_ nanoparticles, similar Ms values (between 12 and 32 emu/g) to our results have been reported in other works of literature [45,46,47].

The magnetization curve shows a narrow hysteresis loop with low coercivity (Hc) and low remanent magnetization (Mr), as seen in the inset of Figure 4. The values of Hc and Mr were 12 Oe and 1.3 emu/g (MgFe_2_O_4_-H&R), as well as 18 Oe and 2.5 emu/g (MgFe_2_O_4_-MW). These are relatively small values that indicate the soft magnetic nature of the synthesized particles at room temperature [48]. Narrow hysteresis loops also indicate that the prepared samples can be easily demagnetized as well.

### 2.6. pDNA Isolation with Amine-Functionalized Magnesium Ferrite Nanoparticles

The *Escherichia coli* (*E. coli)* bacterium was used as a model organism to provide pDNA for the study of reversible DNA-binding properties of the tested MNPs. The isolation procedure is described in Section 3.5. and was performed at least 3 times with the MgFe_2_O_4_-H&R MNPs, as well as with the MgFe_2_O_4_-MW MNPs. All our tests have proved that the MNPs reversibly bind DNA molecules and are able to extract DNA from crude cell lysates. DNA concentrations and the purity of the isolated solutions were determined with micro-volume UV-VIS spectrophotometry.

Both MNPs (MgFe_2_O_4_-H&R MNP and MgFe_2_O_4_-MW MNP) provided similar DNA isolation yields. The mean DNA concentrations isolated with the MgFe_2_O_4_-H&R MNP were 500.83 ± 250.37 µg/mL in the first elution (E1) and 141.13 ± 87.21 µg/mL in the second elution fraction (E2), while in the case of the MgFe_2_O_4_-MW MNP, the isolated DNA yields were 416.80 ± 285.35 µg/mL (E1) and 180.13 ± 16.97 µg/mL (E2), respectively. The appropriate purity values (A_260nm_/A_280nm_) were as follows: 1.93 for E1 and 1.83 for E2 in the case of the MgFe_2_O_4_-H&R MNP-isolated pDNA, and 1.92 (E1) and 1.95 (E2) for the MgFe_2_O_4_-MW MNP pDNA isolation. These values show that the pDNA extracted with the help of both types of amine-functionalized magnesium ferrite nanoparticles can be considered pure, as the values are in the range of 1.7–2.0 [28].

**Figure 5:**
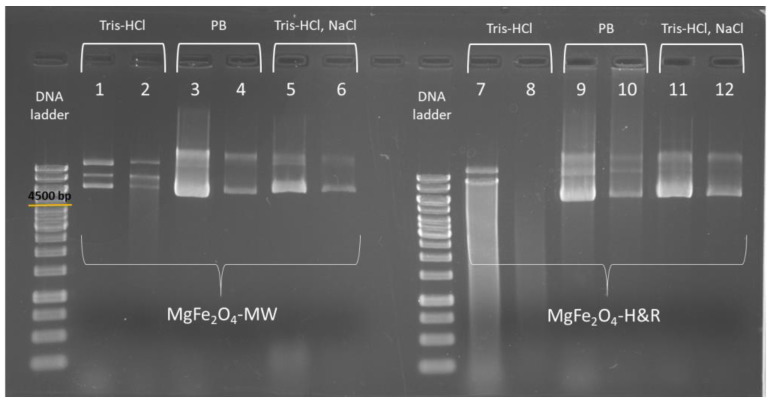
Agarose gel electrophoresis picture of the DNA samples isolated with magnesium ferrite magnetic nanoparticles, differing in the elution buffer. pDNA extracted with MgFe_2_O_4_-MW and MgFe_2_O_4_-H&R MNPs are shown in columns 1–2 and 7–8 containing the first (1 and 7) and second elutions (2 and 8) performed with Tris-HCl (pH 7.0) buffer. Columns 3, 9, 4 and 10 stand for the first and second elutions obtained with phosphate buffer (PB, pH 8.0), while columns 5, 11, 6 and 12 show the first and second elutions using Tris-HCl (pH 8.5) buffer containing 800 mM of NaCl.

Figure 5 shows the agarose gel electrophoresis results of the multiple DNA isolations, where columns 1 and 2 stand for the elution fractions isolated with the MgFe_2_O_4_-MW MNP, while columns 7 and 8 are the elution fractions isolated with the MgFe_2_O_4_-H&R MNP. Additional fluorescent bands (e.g., in columns 1, 2 and 7) show different pDNA conformations [49]. The mean DNA quantity of these samples (where the binding buffer contained NaCl and elution buffer contained only Tris-HCl) were the following: 51.36 µg for the MgFe_2_O_4_-H&R MNP and 47.75 µg for the MgFe_2_O_4_-MW MNP. Smeared bands were found in the case of the MgFe_2_O_4_-H&R MNP gel experiment (Figure 5), which may originate from the residual contamination of the buffers used during isolation. In contrast to this, the isolation performed using the MgFe_2_O_4_-MW MNP resulted in well-defined bands. In addition, it was here that the purity values were also in the required range, with values of 1.92 in E1 and 1.95 in E2. These facts confirm that the quality of the isolated pDNA is already good when using the MgFe_2_O_4_-MW MNP. To further improve the quality of the purified DNA, the composition of the binding and elution buffers was modified, and the corresponding results for the MgFe_2_O_4_-MW MNP are shown in columns 3–6 of Figure 5. The same strategy was applied to eliminate the previously identified faint band from the MgFe_2_O_4_-H&R MNP isolation as well, and columns 9–12 represent the isolations performed using the MgFe_2_O_4_-H&R MNP using the PB, Tris-HCl and NaCl buffers for better isolation.

As seen in Figure 5 (columns 3–4 and 9–10), using 0.1 M potassium phosphate (pH 8.0) as the elution buffer, the DNA desorption from the MNPs (elution step) was successful. This indicates that the negatively charged DNA molecules could be replaced with phosphate ions on the surface of positively charged ferrite MNPs [50]. Xu et al. [18] reported that decreasing the NaCl concentration in the binding buffer and increasing it in the elution buffer can increase the yield of DNA extraction. Based on this, we performed the extraction protocol by removing the NaCl from the binding buffer and adding it to the Tris-HCl elution buffer (final concentrations: Tris-HCl 10 mM (pH 8.5), NaCl 800 mM). These modified buffers resulted in well-defined bands in the gel electrophoresis experiment, as seen clearly from columns 5–6 and 11–12 of Figure 5.

**Figure 6:**
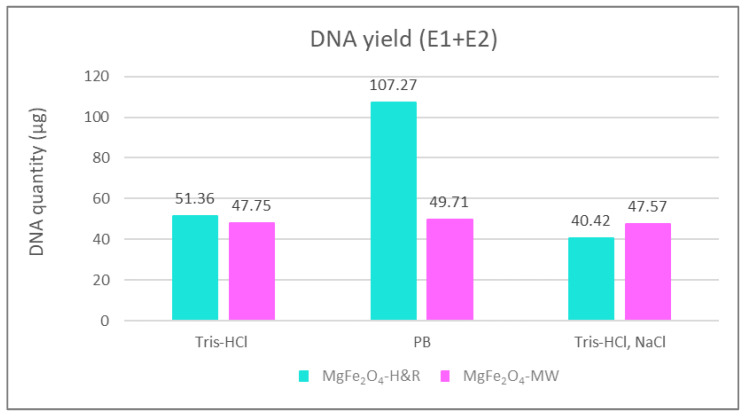
Comparison of the quantity of isolated DNA summarized from the first (E1) and second (E2) elution steps with 3 different elution buffers. DNA purity of the samples (A_260nm_/A_280nm_ value): Tris-HCl as elution buffer with MgFe_2_O_4_-MW (1.94), with MgFe_2_O_4_-H&R (1.88); phosphate buffer as elution buffer with MgFe_2_O_4_-MW (1.79), with MgFe_2_O_4_-H&R (1.88); NaCl containing Tris-HCl elution buffer with MgFe_2_O_4_-MW (2.07), with MgFe_2_O_4_-H&R (2.05). E1 + E2 = sum of the DNA quantity extracted from the first (E1) and second (E2) elution steps; PB = phosphate buffer.

The DNA yields obtained using different elution buffers are shown in Figure 6. Such column representations of the isolation efficiency of MgFe_2_O_4_-H&R MNP clearly show a maximum in the DNA yield when the phosphate buffer (PB) was used as the elution solution (107.27 µg of DNA extracted), while when applying the NaCl-containing elution buffer (Tris-HCl and NaCl, seen in the last two columns in Figure 6), the amount of extracted DNA slightly decreased (40.42 µg in the case of MgFe_2_O_4_-H&R and 47.57 µg for the MgFe_2_O_4_-MW MNP isolations). This may occur due to the fact that when DNA binds to the surface of the used amine-functionalized MgFe_2_O_4_ MNPs in low- or no-salt-containing binding buffers, DNA molecules have an elongated coil conformation [48]. This molecular state allows all accessible phosphate groups along the DNA backbone to bind to the positively charged nanomaterials, leading to fewer DNA molecules interacting with the surface of the MNPs. However, using higher salt concentrations during DNA–MNP binding (when the binding buffer contains NaCl and the elution buffer is only Tris-HCl or phosphate buffer), cations reduce the repulsion between the DNA strands, leading to a compact and bent globular state of the DNA molecule. This means that the DNA loading capacity of MNPs expands in high-salt-concentration binding circumstances because the condensed shape of the DNA molecule reduces the number of electrostatic contact points between the DNA and MNP [50].

These experiments highlight the importance of selecting the right buffer combinations for efficient DNA isolation while maintaining DNA quality and integrity, which is crucial for reliable results.

The DNA isolation efficiency of extraction methods using MgFe_2_O_4_-H&R and MgFe_2_O_4_-MW MNPs was compared to that of a commercially available magnetic particle-based kit. Specifically, we tested the Mag-Bind Ultra-Pure Plasmid DNA 96 Kit from Omega Bio-Tek, which is designed to isolate approximately 50 µg of high-copy-number pDNA. Our results, as presented in Figure 6, showed that the DNA yield obtained with amine-functionalized magnesium ferrite MNPs was at least as high as the kit manufacturer’s guarantee, even for low-copy-number pBAD plasmid DNA. With MgFe_2_O_4_-MW MNPs, we achieved a yield of 47.75 µg of DNA with Tris-HCl elution, 49.71 µg with phosphate buffer and 47.57 µg with Tris-HCl+NaCl elution. For the MgFe_2_O_4_-H&R MNPs, we obtained 51.36 µg of DNA with Tris-HCl elution, 107.24 µg with phosphate buffer and 40.42 µg with Tris-HCl+NaCl elution. These findings confirm the suitability of the synthesized nanoparticles for successful DNA isolation experiments.

DNA purity is an equally important feature of the DNA isolation protocol; therefore, the absorbance values were also measured at 260 nm and 280 nm in all cases. Their ratios, the A_260nm_/A_280nm_ values, were within the appropriate range (specific data can be found in the caption of Figure 6), indicating that the purity of the isolated DNA meets the target values. Therefore, despite the slight decrease in the purity observed with NaCl-containing elution buffer (A_260nm_/A_280nm_: 2.05 for the MgFe_2_O_4_-H&R MNP and 2.07 for the MgFe_2_O_4_-MW MNP), the A_260nm_/A_280nm_ ratios fall within the range found in the previous literature, still indicating insignificant levels of contamination [51,52] and reinforcing the overall suitability of our isolation protocol.

### 2.7. Restriction Digestion of the Extracted pDNA

**Figure 7:**
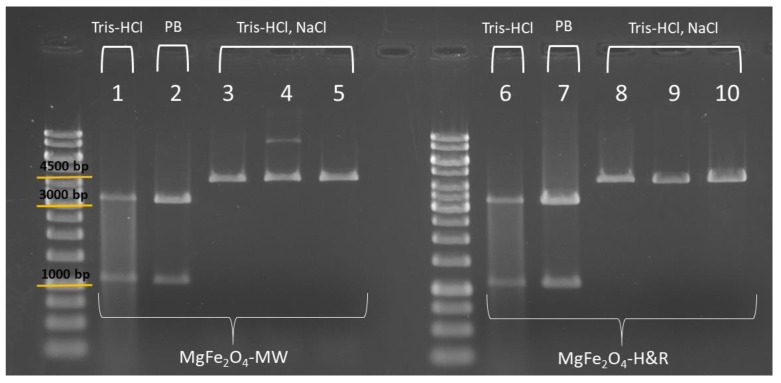
Agarose gel electrophoresis picture of the DNA fragments digested with restriction endonucleases. In the first part of the picture, the isolates of the MgFe_2_O_4_-MW sample are shown, and in the second part, the isolates of the MgFe_2_O_4_-H&R sample (PB = phosphate buffer). In fast digest reactions, two enzymes were used having a single recognition site on the pDNA (1, 2 and 6, 7), while in overnight digestions, one type of linearizing enzyme was used (3, 4, 5 and 8, 9, 10).

To test that the isolated DNA can be a good template for restriction digestion and thus for molecular cloning processes, several different reactions were performed using restriction enzymes from the ThermoFisher Scientific company (Figure 7). In all restriction reactions, pDNA (namely the pBAD24), isolated both with MgFe_2_O_4_-H&R and MgFe_2_O_4_-MW MNPs, was used as a template. The aim of this test was to compare the performance of the templates obtained from the isolation procedures with the MNPs produced by 12-h (H&R) and 4-min (MW) treatments in various digestion reactions. Additionally, we wanted to investigate if there was a difference in the digestion efficiency depending on the elution buffer in which the template pDNA was placed (Tris-HCl only, PB, or Tris-HCl+NaCl).

First, the pDNA samples eluted in Tris-HCl and in phosphate buffer were used as templates in the fast digest reactions. Fast HindIII and Fast VspI (AseI) endonucleases are specific to pBAD24 plasmid DNA, each making a single cut on the double-stranded pDNA. Using them together in the same digestion reaction, two DNA fragments can be obtained (3407 bp (base pair) and 1135 bp, shown in columns 1–2 and 6–7 of Figure 7).

The DNA templates eluted with NaCl-containing buffer (columns denoted as Tris-HCl, NaCl in Figure 7) were investigated in overnight digestions, where conventional restriction digestion buffers can be used. In this case, our aim was to test if extra salt has any effect on the reaction itself since some restriction buffers already contain NaCl (e.g., Red and Orange buffer, according to the manufacturer’s description). Figure 7 also shows the results of the overnight digestions with ClaI (Bsu15I) endonuclease in Tango buffer (columns 3 and 8), HindIII in Red buffer (columns 4 and 9) and VspI (AseI) in Orange buffer (columns 5 and 10). These digestions resulted in a single fragment (linearized pBAD24 plasmid of 4542 bp length), as these enzymes have only one recognition site on pBAD24 (Figure 7, columns 3, 4 and 5: elution fractions of the MgFe_2_O_4_-MW MNP isolations; columns 8, 9 and 10: elution fractions of the MgFe_2_O_4_-H&R MNP isolations). Comparing the agarose gel images of different restriction reactions (where the pDNA in different elution buffers was used as the template), we observed that when using Tris-HCl as the elution buffer (for both the MgFe_2_O_4_-MW and MgFe_2_O_4_-H&R MNP isolations, denoted as columns 1 and 6 in Figure 7), faint restriction fragments were noticeable. However, when digesting the pDNA in the phosphate buffered (PB column) or NaCl-containing elution buffer (Tris-HCl, NaCl columns in Figure 7), the appearance of the fragments on the gel matched the expectations for the restriction digestion results. The presence of additional salts during overnight digestion with the Red or Orange buffers (Figure 7, columns 4, 5, 9, 10) did not negatively impact the restriction reaction. The fluorescent DNA bands appeared at the appropriate height (in line with the 4500 bp marker band) and displayed the expected appearance on the agarose gel.

## 3. Materials and Methods

### 3.1. Materials

The amine-functionalized magnesium ferrite nanoparticles were synthesized from magnesium nitrate hexahydrate, Mg(NO_3_)_2_∙6 H_2_O, MW: 290.79 g/mol (ThermoFisher GmbH, D-76870 Kandel, Germany) and iron(III) nitrate nonahydrate, Fe(NO_3_)_3_∙9 H_2_O, MW: 404.00 g/mol (VWR Int. LtD., B-3001 Leuven, Belgium). Ethylene glycol (EG), HOCH_2_CH_2_OH (VWR Int. Ltd., F-94126 Fontenay-sous-Bois, France), was used as the dispersion medium. For the coprecipitation and functionalization of the ferrites, ethanolamine (EA), NH_2_CH_2_OH (Merck KGaA, D-64271 Darmstadt, Germany), and sodium acetate, CH_3_COONa (ThermoFisher GmbH, D-76870 Kandel, Germany), were used.

The following materials and chemicals were used to maintain the bacterial cell culture and for the plasmid DNA isolation assays with magnetic nanoparticles: tryptone and yeast extract (Neogen Culture Media, Lansing, MI, USA); sodium chloride, bacteriological agar and polyethylene glycol 1450 and 6000 (VWR International, Leuven, Belgium); ampicillin sodium salt (Alfa Aesar, Kandel, Germany); Plasmid Purification Midi Kit (Qiagen, Hilden, Germany); tris hydrochloride salt and bromophenol blue sodium salt (VWR International, Solon, OH, USA); ethylenediamine tetraacetic acid disodium salt (Sigma-Aldrich, Louis, MO, USA); 96% ethanol and glycerol (VWR International, Fontenay-sous-Bois, France); Tween 20, Gel Red nucleic acid gel stain and agarose (Merck Millipore, Billerica, MA, USA); 1 kb DNA ladder (Thermo Fisher Scientific, Waltham, MA, USA).

### 3.2. Synthesis of the Amine-Functionalized MgFe_2_O_4_-H&R by Coprecipitation with 12-h Refluxing

Synthesis of the MgFe_2_O_4_-H&R sample was based on the method of Nonkumwong et al. [31]. In the first step, 1.23 g (15 mmol) of sodium acetate was dissolved in EG (10 mL) and heated to 100 °C during continuous stirring and refluxing for 15 min. A total of 0.2564 g (1 mmol) of magnesium nitrate hexahydrate and 0.8080 g (2 mmol) of iron(III) nitrate nonahydrate were dissolved in 5 mL of EG and then added to the preheated sodium acetate solution. The mixture was stirred for 30 min, then ethanolamine (3.5 mL) was added. The mixture was heated to 200 °C and kept for 12 h in a system with stirring and refluxing. This high viscosity dispersion was cooled down to room temperature; then, it was separated by centrifuging (4200 rpm, 10 min). The solid phase was washed with distilled water several times, and the ferrite was easily separated from the aqueous medium with a magnet. Finally, the ferrite was rinsed with absolute ethanol and dried by lyophilisation.

### 3.3. Synthesis of the Amine-Functionalized MgFe_2_O_4_-MW by Microwave Irradiation-Assisted Coprecipitation

The 12-h heating and refluxing coprecipitation is a protracted and energy-consuming synthesis method. Thus, microwave synthesis was applied for the preparation of amine-functionalized magnesium ferrite. As a result, we significantly shortened the synthesis time to only 4 min from 12 h. During the experiment, the above-mentioned quantities of the metal precursors, sodium acetate, EG and EA, were measured in a PTFE digestion tube (volume: 50 mL). The reaction mixture was treated at 200 °C (350 W) for 4 min at atmospheric pressure in a CEM MDS 81 D microwave digestion instrument. After cooling, the MgFe_2_O_4_-MW sample was separated from the dispersion medium and was washed with distilled water.

### 3.4. Characterization Techniques

High-resolution transmission electron microscopy (HRTEM, Talos F200X G2 electron microscope with field emission electron gun, X-FEG, accelerating voltage: 20-200 kV, ThermoScientific, Waltham, MA, USA) was used for the characterization of the particle size and morphology of ferrite samples. For the imaging and electron diffraction, the SmartCam digital search camera (Ceta 16 Mpixel, 4k x 4k CMOS camera, Thermo Scientific, Waltham, MA, USA) was applied with a high-angle annular dark-field (HAADF) detector. During the sample preparation, the aqueous dispersion of the nanoparticles was dropped onto 300 mesh copper grids (Ted Pella Inc., 4595 Redding, CA 96003, USA). For the quantitative analysis of the magnesium ferrite, the X-ray diffraction (XRD) technique and the Rietveld refinement method were used. For the measurements, a Bruker D8 diffractometer (Cu-Kα source) in parallel beam geometry (Göbel mirror) with a Vantec detector was used. The functional groups on the surface of the amine-functionalized ferrite nanoparticles were identified with Fourier-transform infrared spectroscopy (FTIR) using a Bruker Vertex 70 spectroscope. The measurements were carried out in transmission mode (10 mg of the ferrite sample was pelletized with 250 mg of potassium bromide). The zeta potential of the MgFe_2_O_4_ nanoparticles was examined based on the electrophoretic mobility measurements by applying laser-Doppler electrophoresis with the Malvern Zetasizer Nano ZS equipment (Malvern Panalytical, Malvern, UK). The specific surface area of the samples was measured by CO_2_ adsorption–desorption experiments at 273 K by using a Micromeritics ASAP 2020 sorptometer and the Dubinin–Astakhov (DA) method. The magnetic characterization of ferrite nanoparticles was carried out with a self-developed (University of Debrecen) vibrating-sample magnetometer system based on a water-cooled Weiss-type electromagnet. The powder samples were pelletized for the measurements with a typical mass of 20 mg. The magnetization (M) was measured as a function of the magnetic field (H) up to a 10,000 Oe field strength at room temperature.

### 3.5. pDNA Isolation with Magnesium Ferrite Magnetic Nanoparticles

The pDNA isolation procedure was carried out based on the protocol developed in our previous research on the use of manganese ferrite magnetic nanoparticles in DNA isolation applications [53]. An *E. coli* bacterial cell suspension was used for the isolation of a low-copy-number plasmid DNA called pBAD24. A total of 5 mL of the cells grown in the Luria–Bertani (LB) medium (10 g of tryptone, 10 g of NaCl and 5 g of yeast extract dissolved in 1000 mL of ultrapure water) was centrifuged for 5 min on 6000×g. After decanting the supernatant, cell lysis was carried out by following the Qiagen manufacturer’s recommendations (QIAGEN Plasmid Purification Handbook) using the solutions (P1, P2, P3) prepared as described in the composition description of the QIAGEN Plasmid Purification Midi Kit. In these steps, the macromolecules are precipitated on an alkaline pH value, but by neutralizing the pH, the pDNA is renaturated in the solution. To separate the irreversibly denaturated cell debris and the solution containing the pDNA, a centrifugation step is required (14,500× *g*, 10 min (Mega Star 1.6R centrifuge (VWR International, Leuven, Belgium))). The supernatant fraction was separated. To test the DNA-binding capacity of the amine-functionalized nanoparticles, the separated supernatant was mixed in an Eppendorf tube with 600 µL of 2.3 mg/mL MgFe_2_O_4_ MNPs dispersed in binding buffer (2.5 M NaCl; 1 M Tris-HCl, pH 8.0; 0.5 M EDTA, 20% (*w*/*v*) polyethylene glycol 6000 and 0.05% Tween 20). From this step, the isolation procedure was carried out using a slightly modified version of a protocol from the literature [54]. The DNA–MNP complexes were formed while flipping the Eppendorf tube upside down for 10 min. The cell lysate–MNP complex was incubated at room temperature on a magnetic stand so that the supernatant could be isolated and pipetted into a new tube without disturbing the pellet (DNA–MNP complexes). This was followed by three washing steps with wash buffer containing 1 M Tris-HCl at a pH of 7.5 and 96% ethanol. First, 1000 µL of washing buffer was used, and the dispersion was vortexed and incubated on the magnetic stand for 2 min. The second washing step was shorter, with the same buffer volume; however, this was without vortexing and only 30 s of incubation time. The last step was carried out with 500 µL of washing solution, in which the sample was vortexed and a 2-min incubation was performed on the magnetic stand. To remove the excess ethanol, a short centrifugation step was implemented. This was followed by the incubation of the sample at 37℃ with the tube opened to dry out the magnetic nanoparticles. This is a crucial step that needs to be monitored because an overlong incubation can damage the DNA molecules. Eluting the pDNA from the MNPs is the last phase of the protocol. The DNA–MNP complexes were resuspended in 80 µL of elution buffer (10 mM Tris-HCl, pH 7.0). After a 10-min incubation at 37℃ and 5 min on the magnetic stand, the extracted pDNA was separated from the magnetic nanoparticles. To increase the amount of purified DNA, additional elution steps were implemented.

### 3.6. Gel Electrophoresis

Agarose gel electrophoresis was performed with a Mini-Sub Cell GT horizontal agarose gel electrophoresis system (Bio-Rad Laboratories, Hercules, CA, USA) to confirm the success of the MNP-based DNA extraction. We used 0.75 cm thick 1.0 *w*/*v*% and 0.8 *w*/*v*% agarose gels (1 g or 0.8 g of agarose powder, 100 mL of Tris-Acetate-EDTA buffer (TAE; 40 mM Tris-base, 20 mM acetic acid, 1 mM EDTA)) [55]. TAE buffer was also used as a running buffer for the electrophoresis. To provide the essential density for loading the sample into the agarose gel well and to monitor the process, the 6x gel loading dye solution (1 part DNA loading dye and 5 parts isolated DNA sample) was used (30 *v*/*v*% glycerol, 0.25 *w*/*v*% bromophenol blue dye, and ultrapure water) [55]. The electrophoresis was run for 40 min at 90 V, or in the case of smaller fragments (e.g., ~1100 bp), for 2 h at 30 V.

### 3.7. Quantification of pDNA and Restriction Endonuclease Reactions

The DNA concentration measurements were performed on a NanoDrop One Microvolume UV-Vis spectrophotometer (ThermoFisher Scientific, Waltham, MA, USA). The purity of extracted DNA can be characterized by measuring the absorbance ratio of the 260 and 280 nm wavelengths. For pure DNA solutions, this value should be between 1.7 and 2.0 [28].

The good purity of DNA can also be shown using the purified pDNA in restriction digestion reactions [56]. Different restriction enzymes specific for the pBAD24 plasmid DNA were used to perform fast digestion or overnight digestion reactions. The amount of pDNA used in the restriction reaction was determined from the fluorescence intensity of the sample on the agarose gel and the concentration values obtained from the NanoDrop measurements (varying from 0.5 to 3.5 µg of DNA). The restriction enzyme reaction consisted of 1 µL of Fast Digest or conventional restriction enzyme(s), 2 µL of 10x buffer (Fast Digest, Red, Orange or Tango buffer) and dH_2_O (the final volume of the reaction was 20 µL in all cases). Fast digestion with HindIII and VspI (AseI) endonucleases lasted 10 min at 37 °C, while the inactivation was performed in a 65 °C thermoblock (MultiTherm Shaker (Benchmark Scientific, Edison, NJ, USA)) for 10 min. All the overnight digestions were performed in an incubator (Nuve EN 055 (Nuve Laboratory & Sterilization Technology, Ankara, Turkey)) at 37 °C. The inactivation temperatures differ with enzymes: ClaI (Bsu15I) and VspI (AseI): 65 °C, 20 min; HindIII: 80 °C, 20 min.

## 4. Conclusions

According to the literature, magnesium ferrite magnetic nanoparticles have not been previously investigated using DNA isolation applications. Therefore, in this study, amine-functionalized magnesium ferrite nanoparticles were prepared for application in nucleic acid isolation processes by the coprecipitation synthesis method.

The conventional method involves a 12-h refluxing step (H&R), which is a time- and energy-consuming process; however, this could be shortened to 4 min by using microwave (MW) synthesis, thus offering a faster, more energy-efficient and more effective solution for the synthesis of amine-functionalized MgFe_2_O_4_ particles. Comparing the two above-mentioned synthesis methods, the magnesium ferrite nanoparticles (MgFe_2_O_4_-H&R MNP and MgFe_2_O_4_-MW MNP) showed no significant differences in terms of the crystalline phases, particle morphology, average particle sizes and zeta potentials. Both samples were well-dispersible in aqueous media and were easily demagnetized. These results indicate that both sets of nanoparticles have negatively charged surfaces, suggesting a consistent and comparable surface chemistry.

The two types of MNP samples were compared not only in terms of their physicochemical properties but also in terms of their applicability in DNA purification procedures. Using our former protocol, pDNA from bacterial cells was successfully isolated using both the amine-functionalized MgFe_2_O_4_-H&R and MgFe_2_O_4_-MW MNPs. The DNA quantity values obtained (51.36 µg for the MgFe_2_O_4_-H&R MNP and 47.75 µg for the MgFe_2_O_4_-MW MNP) and the results of restriction digestion confirm the successful isolation. We could conclude that using amine-functionalized MgFe_2_O_4_-H&R or MgFe_2_O_4_-MW MNPs as solid-phase extraction tools provides a DNA yield as good as the yield of commercially available MNP-based pDNA isolation kits [57]. Based on the obtained results in DNA yield, quality and usability in restriction digestion reactions, the nanoparticles synthesized with the shortened procedure (4-min MW synthesis) are as efficient as those produced with the conventional long-term (12-h H&R) procedure. Hence, we propose a new synthesis method for producing good-quality magnesium ferrite particles and a protocol using these MgFe_2_O_4_-MW MNPs as a solid-phase pDNA extraction tool and method for further molecular biological applications.

## Figures and Tables

**Table 1:** Size analysis results of the two types of magnesium ferrite nanospheres that were synthesized using 4-min microwave irradiation (MW) or 12-h heating and refluxing (H&R).

Synthesis Method	Mean	SD	Min.	Max.	1st Quartile	3rd Quartile	Median	P90	P95
	(nm)								
MW	43.9	13.6	17.4	91.3	33.4	53.6	41.2	61.9	68.0
H&R	37.8	8.3	18.5	62.7	32.6	43.6	37.8	47.3	50.9

## Data Availability

The data are available upon request from the corresponding authors.
